# Clinical outcomes of endoscopic resection of preoperatively diagnosed non-circumferential T1a-muscularis mucosae or T1b-submucosa 1 esophageal squamous cell carcinoma

**DOI:** 10.1038/s41598-021-85572-0

**Published:** 2021-03-22

**Authors:** Ken Namikawa, Toshiyuki Yoshio, Shoichi Yoshimizu, Akiyoshi Ishiyama, Tomohiro Tsuchida, Yoshitaka Tokai, Yusuke Horiuchi, Toshiaki Hirasawa, Junko Fujisaki

**Affiliations:** Department of Gastroenterology, Cancer Institute Hospital, Japanese Foundation for Cancer Research, 3-8-31, Ariake, Koto-ku, Tokyo 135-8550 Japan

**Keywords:** Oesophageal cancer, Oesophagogastroscopy

## Abstract

In Japan, preoperatively diagnosed T1a-muscularis mucosae or T1b-submucosa 1 (MM/SM1) esophageal squamous cell carcinoma (ESCC) is a relative indication for endoscopic resection (ER). We evaluated long-term outcomes in patients after ER for non-circumferential ESCC with a preoperative diagnosis of MM/SM1 invasion. We retrospectively reviewed 66 patients with a preoperative diagnosis of non-circumferential MM/SM1 ESCC endoscopically resected between 2010 and 2015. Patients were divided into low- (adequate follow-up) and high-risk (requiring additional treatment) groups for lymph node metastasis according to risk factors (submucosal invasion, lymphovascular invasion, or droplet infiltration) and long-term outcomes were analyzed. Pathological invasion to T1a-lamina propria mucosa, MM/SM1, and T1b-SM2 was seen in 22, 38, and 6 lesions, respectively. Overall, 71.2% patients were classified into the “adequate follow-up” group. Of these, only one patient had a lymph node recurrence, which was successfully treated by additional therapy. The remaining 28.8% patients were classified into the “requiring additional treatment” group, where no recurrences were observed after additional treatments. After a median follow-up of 58.6 months, no deaths happened due to ESCC. The 3- and 5-year overall survival rates were 93.6% and 88.7%, respectively. ER is a valid initial treatment for non-circumferential ESCC with preoperatively diagnosed MM/SM1 invasion.

## Introduction

Esophageal cancer is the seventh most frequent cancer and the sixth most common cause of cancer-related mortality worldwide^1^. It occurs histopathologically in two subtypes, and esophageal squamous cell carcinoma (ESCC) is the most common histologic type of esophageal cancer in the Middle East, Africa, South America, and Asia—including Japan^2^. Despite poor survival rates in patients with advanced-stage ESCC^3,4^, early-stage ESCC can be cured by endoscopic resection (ER), esophagectomy, or chemoradiotherapy (CRT)^5–12^. Several studies have reported favorable outcomes after ER for ESCC^5–10^. The reported rate of lymph node metastasis (LNM) for ESCC—limited to the epithelium (EP) and lamina propria mucosa (LPM) in surgically resected specimens—were 0% and 0–5.6%, respectively^13–16^. Based on these low risks of LNM, cancer predicted to invade into T1a-EP or T1a-LPM (EP/LPM) is a definitive indication for ER according to the guidelines for the diagnosis and treatment of carcinoma of the esophagus, edited by the Japan Esophageal Society (JES)^17^. Based on surgical specimens, once ESCC invades into the muscularis mucosae (MM) or superficial submucosa (SM), the reported rates of LNM limited to MM, the upper third of SM (SM1) and SM deeper than the upper third (SM2) are 5.0–18.0%, 8.3–53.1%, and 22.2–53.9%, respectively^13–16^. Although esophagectomy or CRT is recommended as a first-line treatment for preoperative diagnosis of SM2 cancer because of the high risk of LNM, there is still controversy regarding appropriate treatment of ESCC with preoperative diagnosis of T1a-MM or T1b-SM1 (MM/SM1) invasion. They are relative indications for ER, taking into account those risks of LNM, and we have chosen ER as the first-line treatment for them. However, when more than three-fourths of the esophageal luminal circumference is defected after ER, it can cause stricture in spite of steroid injection or intake and require repeated balloon dilation for several months^18,19^. Therefore, esophagectomy or CRT is recommended for patients with circumferential ESCC in our institution.

The ESCC treatment outcomes in previous reports have relied on pathological results and—although it is important to improve accuracy for diagnosing invasion depth of ESCC—they are not entirely accurate. Thus, it is essential to predict treatment outcomes depending on the clinical diagnosis of invasion depth for improved clinical decision-making and the development of proper treatment strategies.

In the clinical setting, clinicians must decide how best to treat using preoperative information, yet there are very few reports regarding decision-making based on the clinical depth of ESCC invasion^20^. Therefore, this study aimed to describe—in detail—the long-term outcomes of ER for non-circumferential ESCC, based on the preoperative diagnosis MM/SM1 invasion.

## Materials and methods

### Patients

**Figure 1:**
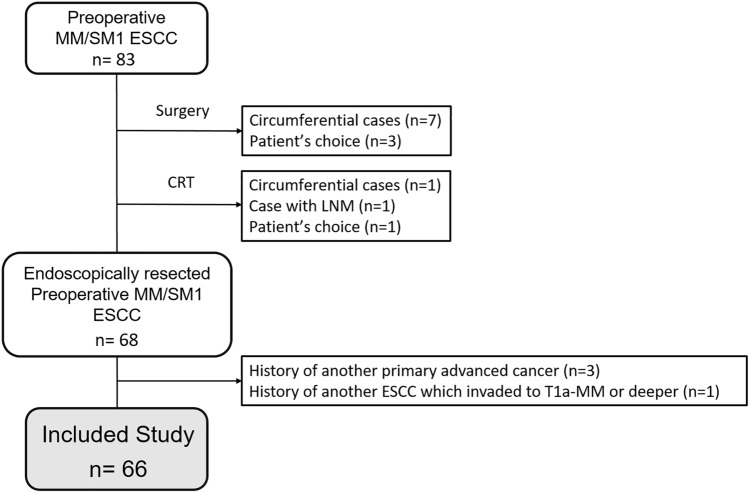
Flow chart of the study subject. *MM* muscularis mucosae, *SM* submucosa, *ESCC* esophageal squamous cell carcinoma, *CRT* chemoradiotherapy.

An initial search of our database identified 83 potential subjects with preoperatively diagnosed MM/SM1 ESCC at our hospital from July 2010 to January 2015. Seventeen patients were excluded according to the following exclusion criteria: (1) treatment with surgery or CRT, (2) history of ESCC that invaded to T1a-MM or deeper, and (3) history of another primary advanced cancer. We retrospectively reviewed remaining 66 patients with ESCC who were preoperatively diagnosed with MM/SM1 invasion and treated by endoscopic mucosal resection (EMR) or endoscopic submucosal dissection (ESD) (Fig. 1). If a patient had more than two lesions preoperatively diagnosed as MM/SM1 cancer, the data from the largest lesion was used for the study. We also reviewed preoperatively diagnosed EP/LPM and SM2 ESCC cases that were endoscopically resected for validation analysis.

### Pretreatment evaluation of clinical invasion depth

**Figure 2:**
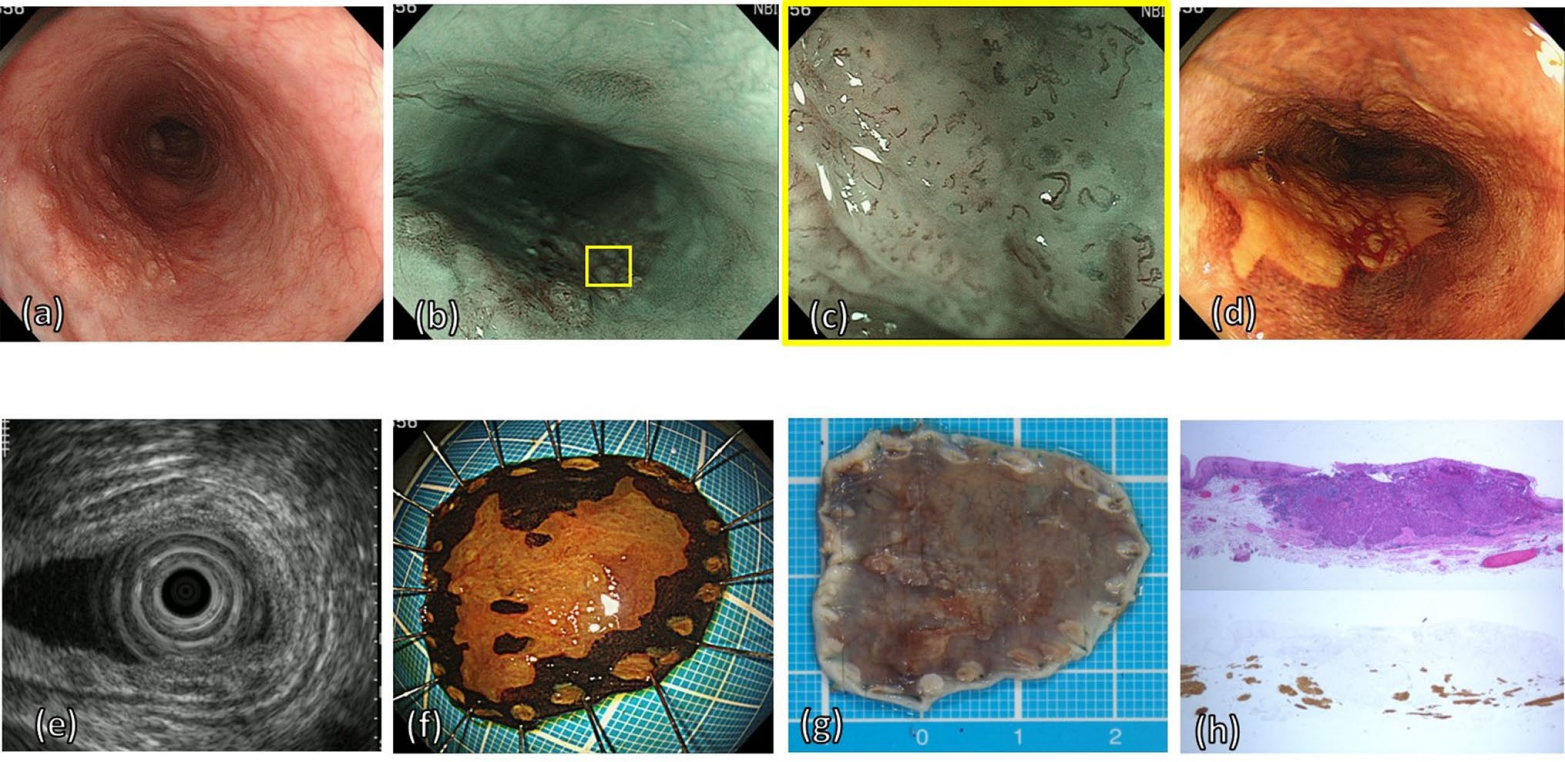
Images of example case of preoperative MM/SM1 ESCC. (**a**) WLI reveals a reddish, superficially elevated lesion with large granular change. (**b**) The lesion appears as a brownish area under NBI. (**c**) Magnifying endoscopy with NBI of the region in the yellow square frame in part (**b**) reveals irregular vessels without loop, the Type B2 vessels, in magnifying endoscopic classification of JES. (**d**) Iodine staining makes it clear to identify the tumor margin as an unstained lesion. (**e**) EUS reveals an irregularity in the first layer, slight thickness in the second layer, and a normal third layer. (**f**) En bloc specimen with iodine staining by ESD. (**g**) The fixed ESD specimen in formalin cut into 2-mm slices. (**h**) HE and desmin staining showing the invasion to MM histopathologically. *MM* muscularis mucosae, *SM* submucosa, *ESCC* esophageal squamous cell carcinoma, *WLI* white light imaging, *NBI* narrow band imaging, *JES* Japan Esophageal Society, *EUS* endoscopic ultrasound, *ESD* endoscopic submucosal dissection, *HE* hematoxylin–eosin.

Endoscopists predicted the invasion depth with morphological findings using conventional white-light imaging (Fig. 2a). In addition, they observed the changes in wall motion by the amount of air sufflation and iodine staining (Fig. 2d). Next, the whole area of the ESCC and, specifically, the area of irregular shape and protrusion were observed by magnifying the endoscopy with narrow band imaging (ME-NBI) (Fig. 2b,c). For lesions in which esophagogastroduodenoscopy (EGD) was performed before 2012, NBI findings were reclassified based on the ME-NBI classification of the JES^21^. Endoscopic ultrasound was performed only for the cases in which the depth of invasion was difficult to predict (Fig. 2e).

As Table 1 shows, the characteristic criteria of EP/LPM were: 0-IIa with small granular change, 0-IIb, shallow 0-IIc with flat surface, and TypeB1 vessels—as per the ME-NBI classification of the JES. The characteristic criteria of MM/SM1 were: 0-IIa with large granular change, thick 0-IIa, shallow 0-IIc with irregular surface, and type B2 vessels. The characteristic criteria of SM2 were: 0-I, 0-IIa with large nodule, obviously thick 0-IIa, deep 0-IIc, 0-IIc with obviously irregular surface, 0-III, and type B3 vessels^22,23^. Thus, endoscopists during preoperative EGD firstly predicted the invasion depth of the ESCC in consultation with experts as necessary. The clinical invasion depth of the ESCC was finally confirmed through a consensus-based discussion during a pretreatment conference.

**Table 1:** Characteristic endoscopic findings of ESCCs according to invasion depth.

	EP/LPM	MM/SM1	SM2
0-I	–	–	Every 0-I
0-IIa	Small granular change	Larger granular change Thickness	Large nodule Obvious thickness
0-IIb	Every 0-IIb	–	–
0-IIc	Shallow depression with flat surface	Shallow depression with irregular surface	Deep depression Obviously irregular su
0-III	–	–	Every 0-III
ME-NBI diagnosis	Type B1	Type B2	Type B3

*ESCCs* esophageal squamous carcinomas, *EP* epithelium, *LPM* lamina propria mucosa, *MM* muscularis mucosae, *SM* submucosa, *ME-NBI* magnifying endoscopy with narrow band imaging.

### Endoscopic resection

ER (EMR or ESD) was performed for patients with preoperative EP/LPM ESCC (Fig. 2f), which is regarded as a definitive indication for ER, according to the Japanese guideline for esophageal cancer treatment^17^. Patients also underwent ER when there was a preoperative diagnosis of MM/SM1 invasion without LNM confirmed by computed tomography scan—regarded as a relative indication for ER in the guidelines—with informed consent being provided for the possibility of additional treatment by way of CRT or operation. We performed esophagectomy or CRT for patients with circumferential preoperative MM/SM1 ESCC and any clinical SM2 ESCC, which are regarded as out of indication for ER.

### Histopathological evaluation and treatment after endoscopic resection

After being fixed in formalin, ER specimens were cut into 2 mm slices (Fig. 2g) and embedded in paraffin. The histopathological type and invasion depth were then evaluated microscopically by pathologists (Fig. 2h). The depth of invasion of the cancer into the submucosa was measured from the deepest MM to the deepest cancer cell nest, as per the Japanese Classification of Esophageal Cancer, 11th edition^24,25^. For the ER specimens, a tumor that had invaded the submucosa within 200 µm from the MM was defined as pT1b-SM1, and a tumor that had invaded deeper was defined as pT1b-SM2. Lymphovascular invasion (LVI) was assessed not only with hematoxylin and eosin (HE) stains but also with D2-40 immunohistochemistry for lymphatic invasion and Victoria blue-HE for venous invasion. LVI was judged positive if vascular or lymphatic invasion was revealed in the resected specimen. Droplet infiltration (DI) is a type of cancer invasion that is reported to be a risk factor for LNM^26,27^ and is determined by the invasion of clusters composed of four or fewer cancer cells.

If pathological diagnosis after ER revealed EP/LPM invasion or pathological T1a-MM invasion without LVI or DI, the patients were considered to be at low-risk of metastasis and were categorized in the “adequate follow-up group.” These patients were provided observation without any additional treatment. If pathological diagnosis after ER revealed pT1b-SM invasion—LVI or DI—the patients were considered to be at high-risk of metastasis and were categorized in the “requiring additional treatment group.” These patients were recommended additional treatment, including esophagectomy and CRT.

### Follow-up

After ER, all patients received standard follow-up, which included EGD with NBI and iodine staining every 6–12 months, and blood tests including tumor marker and computed tomographic scans of the neck, chest, and abdomen, performed every 6 months in patients with pT1a-MM or pT1b-SM invasion for lymph node and distant metastasis screening.

### Statistical analysis

The medians and ranges of all continuous variables are reported. The data were analyzed using GraphPad PRISM (GraphPad Software, Inc., La Jolla, CA, USA). The overall survival estimates were calculated by the Kaplan–Meier method.

### Ethics

This study was designed according to the Helsinki Declaration of the World Medical Association and was approved by the Institutional Review Board of the Cancer Institute hospital of Japanese Foundation for Cancer Research (Approval No. 2016-1053).

### Human rights statement and informed consent

All procedures followed were in accordance with the ethical standards of the responsible committee on human experimentation (institutional and national) and with the Helsinki Declaration of 1964 and later versions. Informed consent or substitute for it was obtained from all patients for being included in the study.

## Results

**Figure 3:**
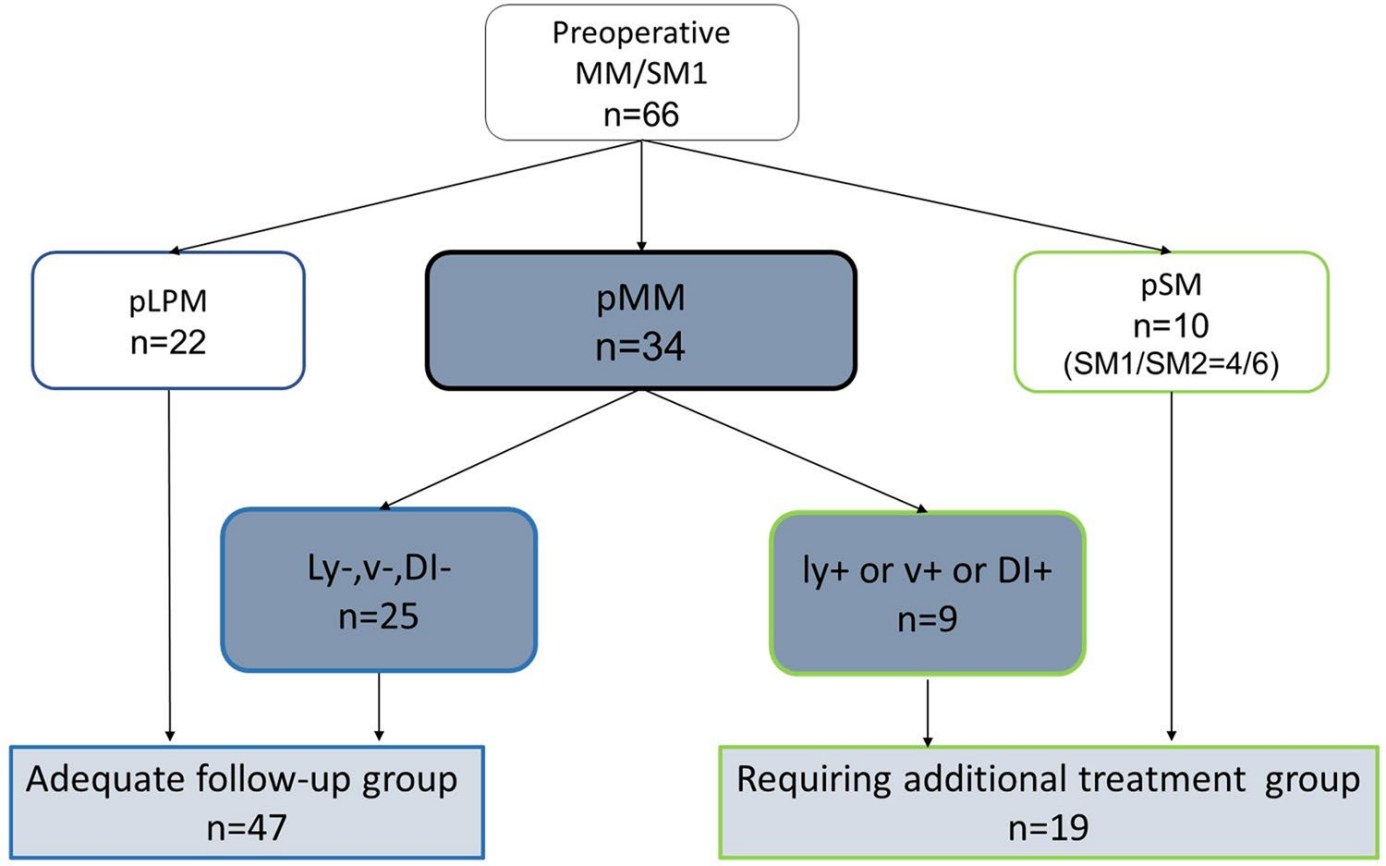
Pathological diagnosis of all preoperative MM/SM1 ESCC. *MM* muscularis mucosae, *SM* submucosa, *LPM* lamina propria mucosa, *DI* droplet infiltration, *ESCC* esophageal squamous cell carcinoma.

The characteristics of the patients and lesions are shown in Table 2. The median age was 67 years (39–81), and the sample included 56 males and 10 females. The median lesion diameter was 24.5 mm (3–70), and 93.0% (66/71) of lesions were resected by ESD. The en bloc resection rates were 100% (5/5) and 97.0% (64/66) by EMR and ESD, respectively. Of the ESD resections, two lesions were resected piecemeal because of technical difficulties due to frequent bleeding and fibrosis after previous ER. There were 5 adverse events, all of which were stricture. Pathological results showed that the positive predictive value (PPV) of diagnosing MM/SM1 invasion was 57.6% (38/66), including 34 pathological T1a-MM and 4 pathological T1b-SM1 cases (Fig. 3).

**Table 2:** Characteristics of patients with preoperative MM/SM1 ESCC and lesion characteristics.

Patients	n = 66
Sex (male/female)	56/10
Median age in years (range)	67.2 (39–81)
Location (Ce/Ut/Mt/Lt/Ae)	2/13/42/9/0
Median size in mm (range)	24.5 (3–70)
Microscopic type (0-I/0-IIa/0-IIb/0-IIc)	0/13/1/52
Luminal circumference (≤ 1/2/1/2 <, < 2/3/2/3 ≤)	39/20/7
EMR/ESD	5/61
En bloc/Piecemeal resection	64/2
Adverse evene (stricture/perforation/postoperative bleeding)	5/0/0

*MM* muscularis mucosae, *SM* submucosa, *ESCCs* esophageal squamous carcinomas, *Ce* cervical esophagus, *Ut* upper thoracic esophagus, *Mt* middle thoracic esophagus, *Lt* lower thoracic esophagus, *Ae* abdominal esophagus, *EMR* endoscopic mucosal resection, *ESD* endoscopic submucosal dissection.

Nine out of 34 pathological T1a-MM cases—including seven LVI-positive, four DI-positive cases, and two both-positive cases—required additional therapy (Fig. 3). The remaining 25 cases did not have LVI or DI, and these patients were recommended observation without additional therapy as adequate treatment. Adding 22 cases of pT1a-LPM to these 25 cases of pathological T1a-MM without any other risk, in total, as many as 71.2% (47/66) of all cases were regarded as belonging to the adequate follow-up group after ER.

The results of the validation analysis with preoperatively diagnosed EP/LPM and SM2 ESCC cases that were endoscopically resected are shown in Supplementary Fig. 1. Of the preoperatively diagnosed EP/LPM lesions, 98.8% (511/517) and 1.2% (6/517) of the lesions were included in the adequate follow-up and requiring additional treatment groups, respectively. Of preoperatively diagnosed SM2 lesions, 10% (1/9) and 90% (9/10) of the lesions were included in the adequate follow-up and requiring additional treatment groups, respectively.

**Figure 4:**
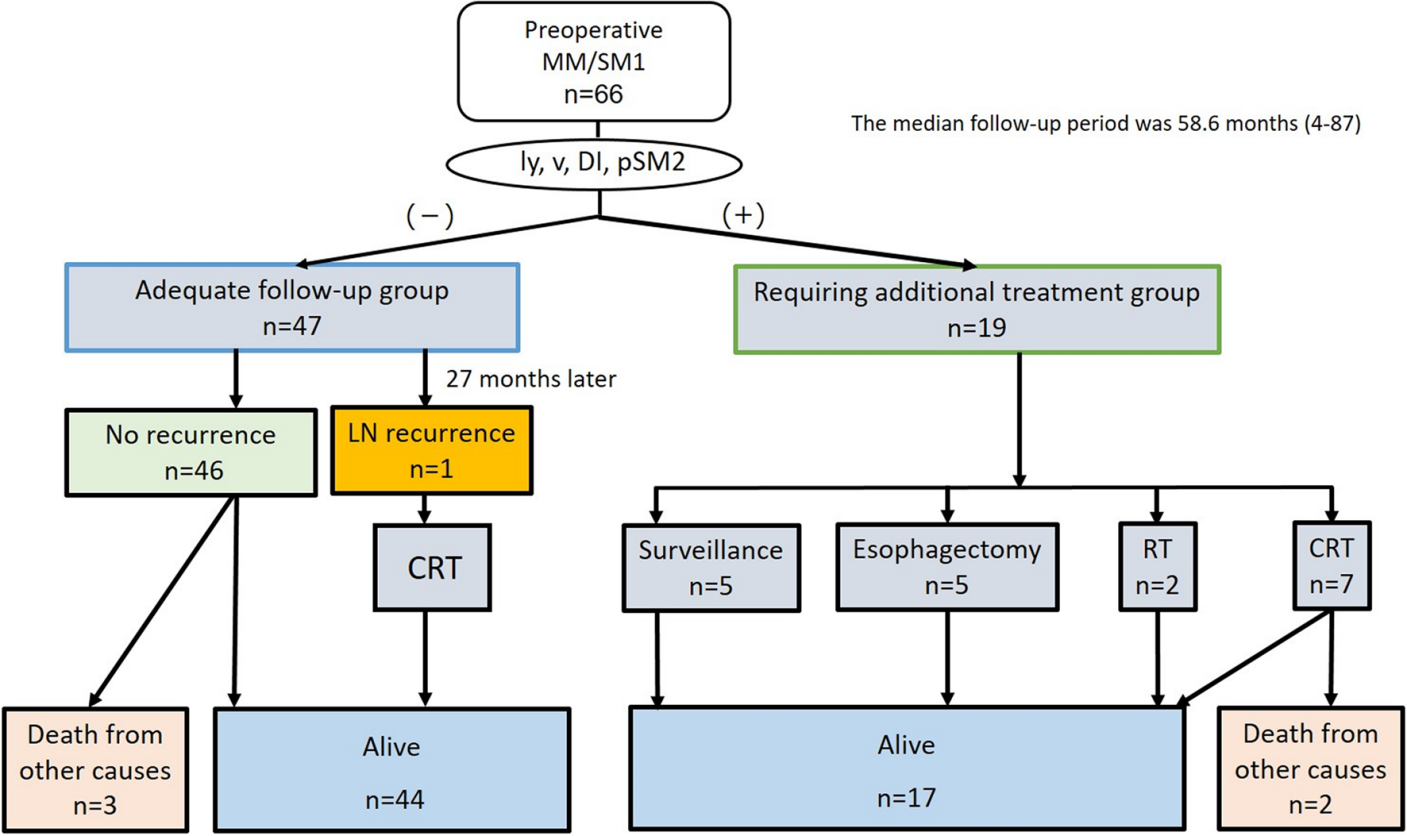
Long-term outcomes of patients with preoperative MM/SM1 ESCC. *MM* muscularis mucosae, *SM* submucosa, *DI* droplet infiltration, *LN* lymph node, *CRT* chemoradiotherapy, *RT* radiotherapy.

**Figure 5:**
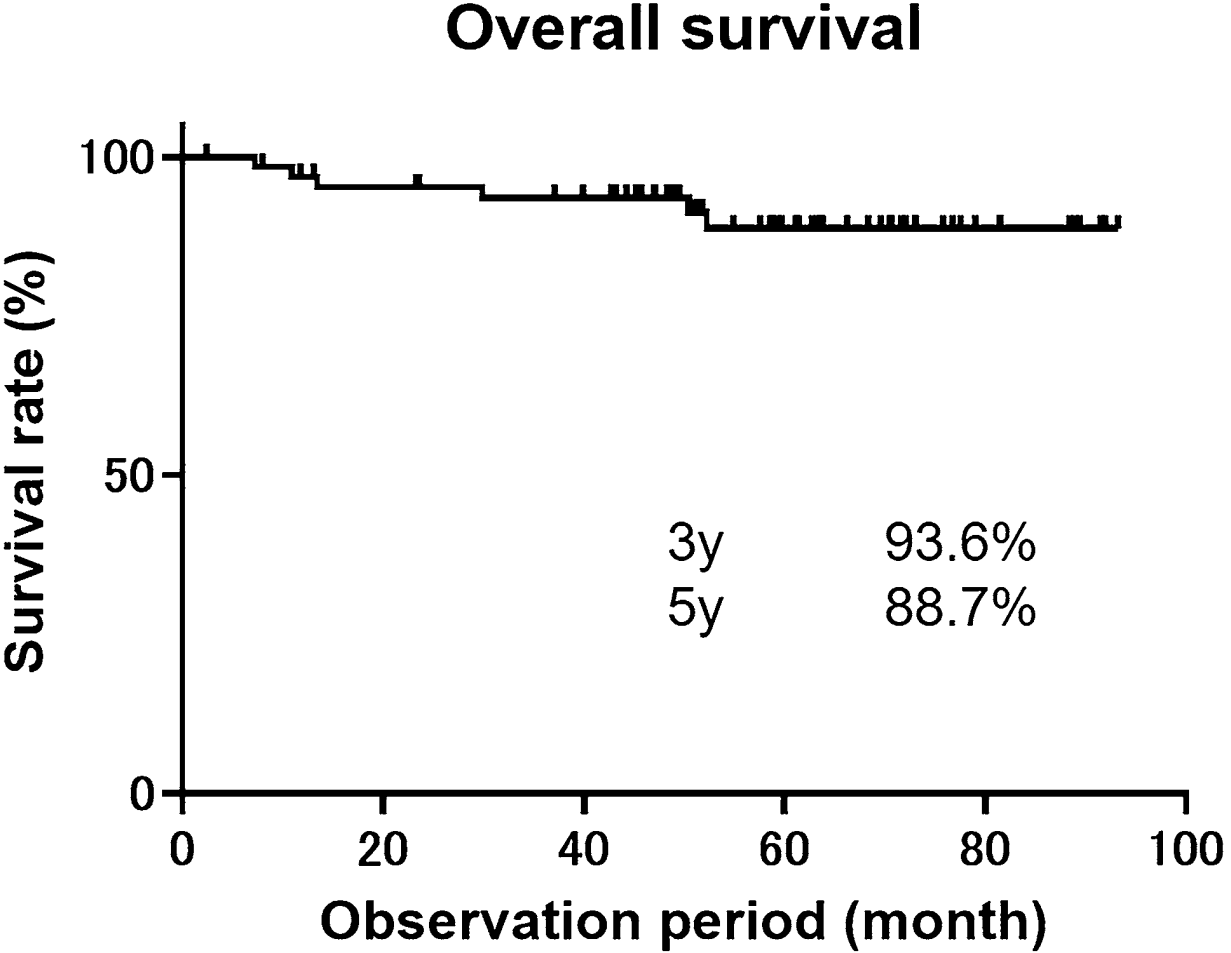
Overall survival of patients with ESCC after ER. *ESCC* esophageal squamous cell carcinoma, *ER* endoscopic resection, *y* year.

The clinical course of patients is shown in Fig. 4. In total, 97.9% (46/47) of patients in the adequate follow-up group had no recurrence, except for one patient who had a lymph node recurrence that was successfully treated. This lesion was treated by en block resection and histology showed 35 mm, 0-IIc, pT1a-MM, resection margin negative, DI negative, and LVI negative; lymph vessel infiltration was revealed in T1a-LPM. This patient was not deceased 36 months after additional CRT. On the other hand, 19 patients with LVI, DI, or SM invasion in the requiring additional treatment group underwent CRT (n = 7), radiotherapy (RT) (n = 2), esophagectomy (n = 5), or elected to be in the surveillance group (n = 5) without any additional treatment (considering the patient’s condition). No recurrence was observed in any of these patients. The median follow-up period was 58.5(4–93) months, during which five patients (7.6%) died. All five patients died due to causes other than ESCC—two due to lung cancer, one due to hypopharyngeal cancer, and two from unknown causes. At the end of the follow-up period, the 3-year and 5-year overall survival rates were 93.6% and 88.7%, respectively (Fig. 5).

## Discussion

In the current study, we retrospectively reviewed 66 patients with preoperatively diagnosed T1a-MM or T1b-SM1 non-circumferential ESCC who were treated by ER to describe their long-term outcomes. Our data showed favorable outcomes after ER for preoperative MM/SM1 non-circumferential ESCC: there was no cause-specific death, with 3- and 5-year overall survival rates of 93.6% and 88.7%, respectively. This is the first report that provides a detailed evaluation of long-term patient outcomes for preoperative diagnosis of MM/SM1 non-circumferential ESCC, treated by ER.

Surgery has generally been the first-line treatment for ESCC with preoperative MM/SM1 invasion, and may remain the preferred choice in some hospitals. However, our results show that it is effective to treat patients with preoperative MM/SM1 invasion by ER because pT1a-MM without LVI occupy the largest portion (65.8%) of all pMM/SM1 lesions, and these tumors have very low risk of metastasis^8,10^. In endoscopically resected pT1a-MM cancer, the rate of LNM has been reported to be only 0–4.2%, which is lower than the those reported in surgical operation cases^8,10,14^. These differences in LNM rate could be due to the method of histopathologic evaluation of the lesions. Compared to 2 mm slices from ER specimens, 5 mm slices from surgical specimens could be underestimated for invasion depth, leading to overestimation of the rate of LNM^10,13,15,16^.

The PPV of diagnosing MM/SM1 was 57.6% in this study, similar to that of previous study^28^. This low PPV is partially because the MM and SM1 only consist of a thin layer (especially SM1 which is ≦ 200 µm), which also means that pT1b-SM1 ESCC is relatively rare. We overestimated pT1a-LPM lesions in 33.3% (22/66) of cases and underestimated pT1b-SM2 in 9.1% (6/66) of all preoperative MM/SM1 lesions. In the cases of underestimation, there were no endoscopic findings to suggest that the tumor was invading the deep submucosa, such as a large nodule, deep depression, or large vessels (Type B3 vessels)^25^. Of these, one tumor had invaded into the deep submucosa only through intraductal invasion, three tumors had invaded areas too small to detect (less than 2 mm), one tumor had invaded a 4 mm area, and the other had invaded a 10 mm area. The 10-mm area with pT1b-SM2 (400 µm) invasion was slightly superficially elevated; however, this was observed retrospectively, and the lesion did not show typical deep submucosal invasion on the endoscopic findings. These underestimated lesions were considered difficult cases in which to diagnose the depths, even upon reanalysis.

There are some preoperative MM/SM1 lesions for which ER may not be suitable. We excluded a circumferential ESCC from the ER criteria. The stricture caused by ER could make patients lose the appropriate timing for additional CRT. The efficacy and safety of ER combined with CRT for treating T1a-MM and T1b-SM ESCC have been reported^29,30^. The favorable outcome of esophagectomy for pT1a-MM with LVI and T1b-SM after ER has also been reported^31^. Therefore, ER should be avoided for lesions with a high risk of stricture if CRT is being considered as an additional treatment. In other words, ER would be a suitable choice for circumferential preoperative MM/SM1 ESCCs if esophagectomy is the patient’s choice for the additional therapy, or if the patient cannot undertake CRT due to a concurrent disease such as renal dysfunction, thrombocytopenia due to idiopathic thrombocytopenic purpura, or liver cirrhosis. There were 5 adverse events of stricture in the current study. It occurred in 2 ESCC cases with more than half and less than two thirds circumference and in 3 ESCC cases with two thirds or more of circumference, all of which were successfully managed by several balloon dilations. There were no other adverse events such as perforation or post bleeding.

Thus, we suggest the indications for ER for preoperative MM/SM1 should be both lesions without a high risk of stricture—non-circumferential lesions—and any lesions for which esophagectomy is planned as additional therapy. Furthermore, ESD is a better choice than EMR to achieve en bloc resection—especially for larger lesions, because accurate histological evaluation is essential for predicting the risk of LNM. Surgical operation or definitive CRT might be the better option than piecemeal ER in an institution where ESD cannot be performed safely.

In the present study, 71.2% (47/66) of patients were not recommended any additional therapy. This included 25 cases of pathological T1a-MM without any other risk factors, and 22 cases of pT1a-LPM. This favorable result stems from two reasons. Firstly, 73.5% (25/34) of all pT1a-MM tumors did not have LVI or DI for LNM and pT1a-MM tumors occupied 87.5% (34/38) of pathological MM/SM1 tumors. Secondly, we tended to overestimate MM/SM1 ESCCs under our diagnostic criteria—more pT1a-LPM tumors (33.3%) were overestimated as preoperative MM/SM1 than pSM2 tumors (9.1%) underestimated as preoperative MM/SM1. Moreover, the rest of all cases—27.3% (19/66)—with high risk factors for metastasis, were treated appropriately with additional therapy, considering patient risk. These results led to no cause-specific deaths and good prognosis in terms of the 5-year survival rate. Thus, we consider preoperative MM/SM1 non-circumferential ESCC as an adequate target for ER treatment.

Validation analysis showed that among the endoscopically resected ESCC, 98.8% (511/517) of preoperatively diagnosed EP/LPM ESCC and 10% (1/10) of preoperatively diagnosed SM2 ESCC were classified into the adequate follow-up group. This result suggested that it was unquestionable to regard preoperatively diagnosed EP/LPM as a definitive indication for ER, and preoperatively diagnosed MM/SM1 ESCC had a much better indication to be treated by ER than preoperatively diagnosed SM2 ESCC.

Our study had several limitations. First, this was a single center retrospective study, which limits the generalizability of the results. Second, the number of preoperative MM /SM1 tumors was limited because of low frequency; however, the data was sufficient for analyzing cause-specific survival and overall survival rates in the adequate follow-up group after ER. Third, some of the patients in the surveillance group—despite recommendations—refused additional treatment, which were exceptional cases. However, we think this made us consider the practical outcome of patients with preoperative MM/SM1 ESCC, including outcomes associated with deviation from treatment criteria.

## Conclusion

Patients who underwent ER for preoperatively diagnosed non-circumferential T1a-MM or T1b-SM1 ESCC with an appropriate additional therapy if needed had favorable treatment outcomes. No requirement for additional treatment in 71.2% of the cases, only 1 case showed LN recurrence, and there were no ESCC related deaths. Our study suggests that ER could be valid as initial treatment for non-circumferential ESCC with a predicted preoperative invasion depth of MM/SM1.

## Supplementary Information

Supplementary Legend.

Supplementary Figure S1.
