# Author Correction: Clinical outcomes of endoscopic resection of preoperatively diagnosed non-circumferential T1a-muscularis mucosae or T1b-submucosa 1 esophageal squamous cell carcinoma

**DOI:** 10.1038/s41598-021-03798-4

**Published:** 2021-12-14

**Authors:** Ken Namikawa, Toshiyuki Yoshio, Shoichi Yoshimizu, Akiyoshi Ishiyama, Tomohiro Tsuchida, Yoshitaka Tokai, Yusuke Horiuchi, Toshiaki Hirasawa, Junko Fujisaki

**Affiliations:** Department of Gastroenterology, Cancer Institute Hospital, Japanese Foundation for Cancer Research, 3‑8‑31, Ariake, Koto‑ku, Tokyo, 135‑8550 Japan

Correction to: *Scientific Reports* 10.1038/s41598-021-85572-0, published online 22 March 2021

The original version of this Article contained an error in the Materials and methods, under the subheading ‘Histopathological evaluation and treatment after endoscopic resection’,

“LVI was judged positive if vascular or lymphatic invasion was revealed in the MM or deeper layer. Droplet infiltration (DI) is a type of cancer invasion that is reported to be a risk factor for LNM^26,27^ and is determined by less than four cancer cells invading (apart from the main advanced part).”

now reads:

“LVI was judged positive if vascular or lymphatic invasion was revealed in the resected specimen. Droplet infiltration (DI) is a type of cancer invasion that is reported to be a risk factor for LNM^26,27^ and is determined by the invasion of clusters composed of four or fewer cancer cells.”

The original Article has been corrected.

