# Supplementary material for: Clinical outcomes of endoscopic resection of preoperatively diagnosed non-circumferential T1a-muscularis mucosae or T1b-submucosa 1 esophageal squamous cell carcinoma
Source: Sci Rep. 2021 Mar 22;11:6554. doi: 10.1038/s41598-021-85572-0 (PMC7985298; doi:10.1038/s41598-021-85572-0)
Supplement: Supplementary file 1 — Supplementary Legend. [file 41598_2021_85572_MOESM1_ESM.docx]

**Supplementary material**

**Title**

**Clinical outcomes of endoscopic resection of preoperatively diagnosed non-circumferential T1a-muscularis mucosae or T1b-submucosa 1 esophageal squamous cell carcinoma**

**Authors**

**Ken Namikawa, MD, Toshiyuki Yoshio* MD, PhD, Shoichi Yoshimizu, MD, Akiyoshi Ishiyama, MD, Tomohiro Tsuchida MD, PhD, Yoshitaka Tokai, MD, Yusuke Horiuchi MD, PhD, Toshiaki Hirasawa, MD, Junko Fujisaki MD, PhD**

**Department of Gastroenterology, Cancer Institute Hospital, Japanese Foundation for Cancer Research, Tokyo, Japan**

**Supplementary Figure Legends**

**Supplementary Fig. 1.** Outcome of classifying preoperative EP/LPM and SM2 ESCC cases after ER based on the risk of LNM

Abbreviations: EP, epithelium; LPM, lamina propria mucosa; SM, submucosa; ESCC, esophageal squamous cell carcinoma; ER, endoscopic resection; LNM, lymph node metastasis
