# Supplementary figures and images for: Clinical outcomes of endoscopic resection of preoperatively diagnosed non-circumferential T1a-muscularis mucosae or T1b-submucosa 1 esophageal squamous cell carcinoma
Source: Sci Rep. 2021 Mar 22;11:6554. doi: 10.1038/s41598-021-85572-0 (PMC7985298; doi:10.1038/s41598-021-85572-0)

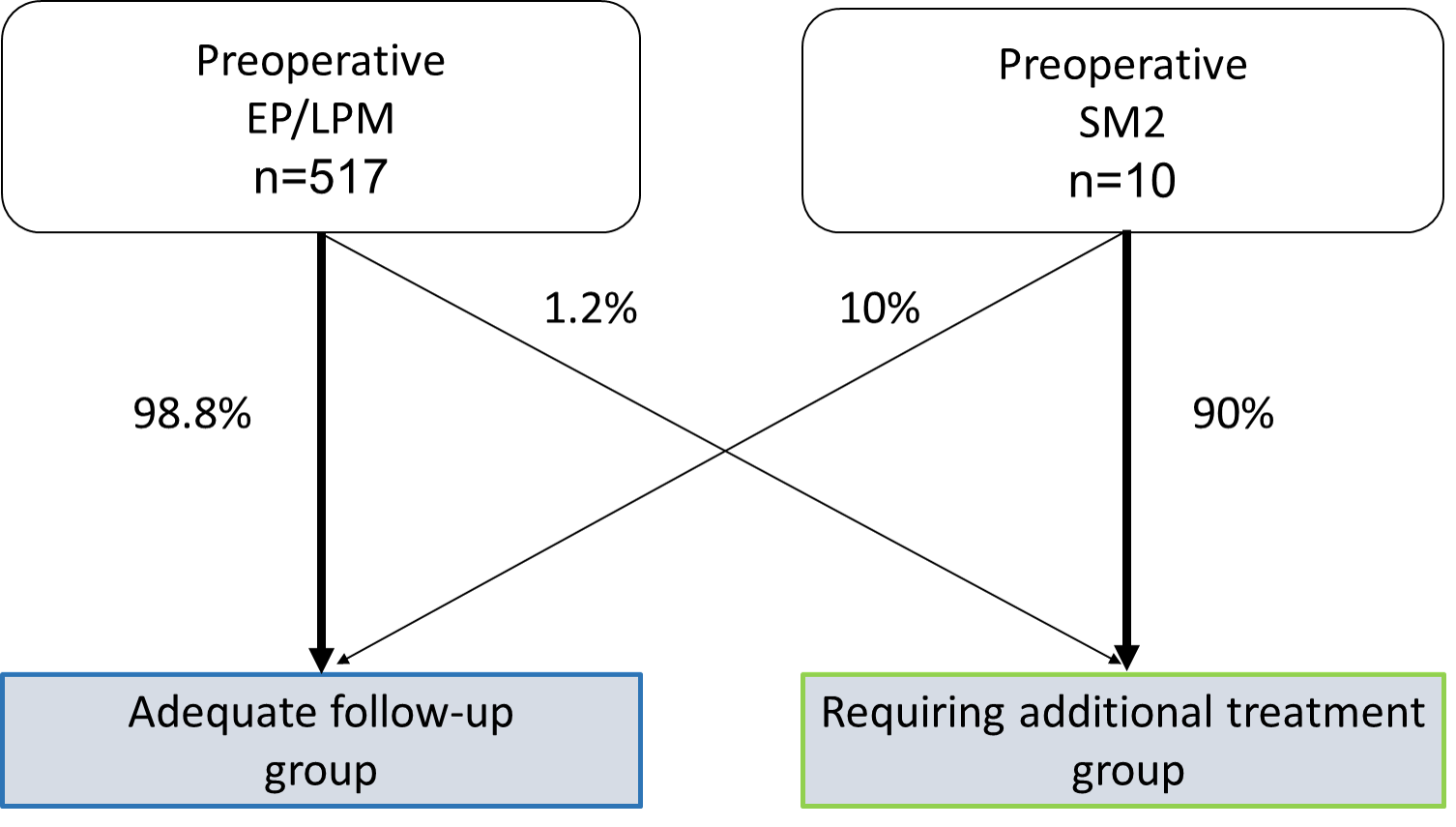

Supplement: Supplementary file 2 — Supplementary Figure S1. [file 41598_2021_85572_MOESM2_ESM.tif]
